# Nano carbon-modified air purification filters for removal and detection of particulate matters from ambient air

**DOI:** 10.1038/s41598-023-50902-x

**Published:** 2024-01-05

**Authors:** Yasser A. Attia, Abd Elhakim Ezet, Samar Saeed, Ahmed H. Galmed

**Affiliations:** 1 National Institute of Laser Enhanced Sciences, Cairo University, Giza, 12613 Egypt; 2 Air Quality Lab, Cairo University Center for Hazard Mitigation, Giza, 12613 Egypt

**Keywords:** Climate sciences, Environmental sciences, Natural hazards, Health care, Nanoscience and technology

## Abstract

Particulate matters (PMs) pose significant risks to human health and the environment, necessitating research to enhance air purification filters and reduce harmful emissions. This study focuses on the preparation of carbon nanomaterials, including graphitic carbon nitride nanosheets (g-C3N4 NSs), reduced graphene oxide (r-GO), and carbon nanotubes (CNT), for modifying filters in air particle monitoring devices. The objective is to investigate the impact of these nanomaterials on enhancing PM adsorption efficiency. Quantitative and qualitative analyses of the modified filters’ adsorption efficiency towards PMs are performed using spectroscopic techniques such as Energy-Dispersive X-ray Spectroscopy (EDX), Inductively Coupled Plasma (ICP), and Laser-Induced Breakdown Spectroscopy (LIBS). The results reveal that CNT-modified filters exhibit superior adsorption efficiency compared to the control, g-C3N4, and r-GO-modified filters. The exceptional performance of CNTs is attributed to their large specific surface area and pore volume. Additionally, LIBS demonstrates its capability to detect heavy metals like Cd, which remain undetected by EDX and ICP. The technique proves sensitive for heavy metal monitoring. This novel approach is expected to garner significant attention and contribute to the development of improved air purification technologies.

## Introduction

Air pollution is a complex global issue that poses significant hazards to human health, ecosystems, and the Earth’s climate system. It encompasses the presence of various chemical substances or physical effects in the atmosphere, either temporarily or permanently, that have adversative properties on human well-being, comfort, the food chain, property values, and the overall stability of ecosystems. Air pollutants can exist in the form of liquids, gases, or solid particles, ranging from massive to sub-molecular sizes. They can originate from both natural sources, such as volcanic eruptions and forest fires, and human-made activities, including industrial processes, transportation, and energy production. Even though anthropogenic sources account for less than 0.01% of the global air composition, their influence can have far-reaching consequences on the environment, climate, and the living organisms inhabiting the planet^1,2^. The impact of air pollutants on the environment and human health cannot be underestimated. Even slight alterations in the atmospheric composition can have profound adverse effects on climate patterns, disrupt ecosystems, and threaten the survival of numerous species. Examples of such impacts include the occurrence of acid rain, the presence of ozone in the lower atmosphere, and the formation of photochemical smog^3,4^. These pollutants, whether visible or invisible, comprise gases or particles that are not natural components of the air. While natural sources like pollen, dust storms, and forest fires contribute to these pollutants, an even greater concern arises from the emission of pollutants from human activities. The combustion of fossil fuels, the burning of coal, wood, and other fuels in vehicles, homes, and factories, all release pollutants into the air, contributing to the degradation of air quality^5–8^.

Particulate matter (PM) occupies a prominent position among the major pollutants in the atmosphere. It is considered one of the six criteria air pollutants classified by the U.S. Environmental Protection Agency (USEPA). PM is a term that describes a mixture of particles of various shapes and sizes that enter the atmosphere from a variety of sources, including the combustion of fossil fuels, industrial emissions, dust, smoke, and fog. These particles can range from coarse to fine, with diameters varying from fewer 10 µm to sub-micrometres^9^. Particulate matter poses a significant risk to human health as it can penetrate deep into the respiratory system when inhaled, leading to respiratory infections and exacerbating heart and lung diseases. Additionally, it is well-recognized that PM particles affect the climate system, affecting cloud development, the Earth’s radiation budget, and acting as catalysts for air pollution in the upper atmosphere. The harmfulness of particulate matter is strongly correlated with its size. Particulate matter with a size smaller than 10 µm, referred to as PM10, poses the highest level of risk due to its ability to deeply penetrate the lungs and potentially enter the bloodstream. The health impacts associated with PM10 include respiratory and cardiovascular problems. Furthermore, scientific studies have linked exposure to particle pollution to a range of health issues, such as asthma, bronchitis, lung cancer, and premature death. The most vulnerable groups to the effects of particle pollution include young children, older individuals, and those with pre-existing heart or lung conditions^10,11^.

In recent years, nanoparticles have gained significant attention in various fields, including engineering, electronics, and medicine. These materials exhibit unique physicochemical properties that are distinct from their macroscopic counterparts. Nanoparticles possess different atomic and electronic structures, and a higher relative fraction of surface atoms compared to bulk materials. This increased surface area-to-volume ratio contributes to their enhanced reactivity and the emergence of novel properties that can be harnessed for various applications^12–15^. The application of nanoparticles in air pollution remediation has shown promising potential. These materials offer high reactivity, efficient pollutant adsorption, and cost-effectiveness compared to conventional strategies. Nanoparticles have demonstrated their ability to adsorb atmospheric heavy metals, contributing to the reduction of heavy metal pollution. Moreover, engineered nanoparticles have been utilized to clean indoor air pollutants effectively. Nanotechnology has thus opened new possibilities for tackling air pollution issues, providing hope for improved air quality and human well-being^16–19^.

Heavy metal pollution has become a major environmental concern in recent years due to their toxicity, bioaccumulation, and persistence in the environment. Traditional methods for removing heavy metals from water, such as chemical precipitation and ion exchange, have limitations such as high cost, low efficiency, and generation of secondary pollutants. Carbon-based nanomaterials (CBNMs) have emerged as promising alternatives for heavy metal removal due to their unique properties, including: (i) High surface area: CBNMs like carbon nanotubes (CNTs) and graphene have extremely large surface areas, providing a large number of binding sites for heavy metal ions^20^. (ii) Tunable surface chemistry: CBNMs can be readily modified with various functional groups, such as oxygen-containing groups, to enhance their selectivity and affinity for specific heavy metals. (iii) High mechanical strength and chemical stability: CBNMs are resistant to degradation and can be reused multiple times, making them a cost-effective and sustainable solution^21^. (iv) Good adsorption capacity: CBNMs can bind to a wide variety of heavy metals with high efficiency and selectivity^22^. Several types of CBNMs have been investigated for heavy metal removal, including Carbon nanotubes (CNTs): CNTs have a high surface area and can be modified with various functional groups for specific heavy metal removal. Graphene and graphene oxide (GO): Graphene and GO have large surface areas and abundant oxygen-containing groups, making them effective for adsorbing heavy metals. Graphitic carbon nitride (g-C_3_N_4_): AC is a porous material with a high surface area and is widely used for adsorption of various contaminants, including heavy metals.

By presenting the previous work in the scope of the use of carbon-based nanomaterials and their applications in the field of reducing environmental pollution in general and reducing air pollution specially, the aim of this work is to prepare CNTs, r-GO, and g-C_3_N_4_ nanomaterials as examples of CBNMs to modify the filters that operated by air particle monitoring devices and study the impact of CBNMs on increasing the efficiency of filter adsorption of PMs and improve the PMs monitoring spectroscopic techniques.

## Results

**Figure 1:**
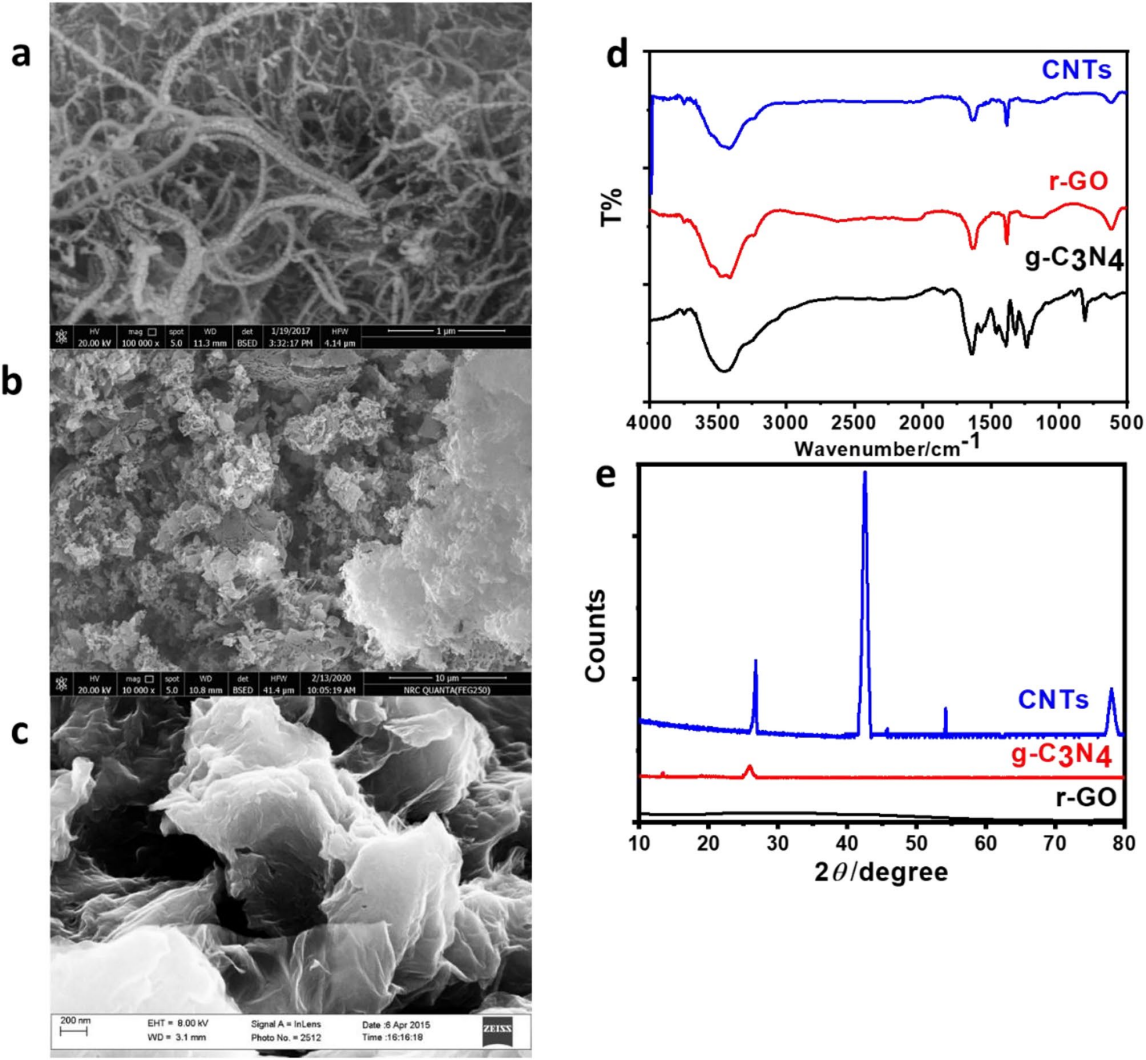
SEM images of the prepared carbon nanotubes (**a**), g-C_3_O_4_ (**b**), and r-GO (**c**), respectively, FTIR spectra of the prepared CBNMs (**d**), and XRD patterns of the prepared CBNMs (**e**).

CNTs were obtained showing diameters ranging from 40 to 50 nm and lengths of approximately 20 mm, as depicted in the electron microscope images shown in Fig. 1A. The pristine g-C_3_N_4_ exhibited typical layered and stacked structures, as observed in the SEM image presented in Fig. 1B. This sample comprised nano-sheet-like structures and appeared fluffier. On the other hand, the r-GO nanosheets displayed irregular and folding layer structures, as shown in the SEM image in Fig. 1C. These nanosheets were entangled with each other, and Fig. 1C also revealed that the single- or few-layer r-GO nanosheets exhibited numerous wrinkles.

The FTIR spectrum of raw CNTs in Fig. 1d exhibited a broad absorption peak in the range of 3450–3460 cm^−1^, corresponding to the –OH group, indicating the presence of hydroxyl groups on the surface of the CNTs. The peaks at 2950 and 2850 cm^−1^ corresponded to the C–H stretch vibration, while the C–C characteristic peak was observed at 1580 cm^−1^. Another peak at 1650 cm^−1^ represented the C–O stretching mode of functional groups on the surface of multi-walled carbon nanotubes (MWCNTs) or the absorption of atmospheric CO_2_ on the composite surface. The peak at 950 cm^−1^ could be assigned to the C–O stretching mode. The stretching modes of CN heterocycles connected to the skeletal stretching vibrations of aromatic rings were represented by the peaks at 1145, 1213, 1393, 1587, and 1648 cm^−1^ in the FTIR spectra of g-C_3_N_4_. The triazine units of g-C_3_N_4_ were in the breathing mode as shown by the peak at 810 cm^−1^ (Fig. 1d). In the case of graphene, the three bands 1724, 1222, and 1050 cm^−1^ attributed to carbonyl, epoxy, and alkoxide functional groups, respectively, were significantly reduced compared to those of GO, indicating the deoxygenation of the sheets, as shown in Fig. 1d.

In Fig. 1e, the XRD pattern of CNTs displayed characteristic diffraction peaks at 26.52°, 42.48°, 54.71°, and 78.43° 2θ, corresponding to the (220), (100), (004), and (110) reflection planes, respectively^23^. The XRD pattern of pure-g-C_3_N_4_ exhibited peaks at 26.73° and 13.37° 2θ, which could be assigned to the (002) inter-layer structural packing crystal plane and (100) inter-planar stacking diffraction planes, respectively. The strong peak at 26.73° indicated the stacking reflection of conjugated aromatic systems, revealing a graphitic structure with an interlayer distance of 0.326 nm, as illustrated in Fig. 1e^15^. The three-dimensional character of graphene oxide was reduced, as evidenced by the absence of the narrow XRD reflection at 2θ = 10.8 A°. Instead, a broad band was observed in the case of r-GO, possibly due to intra-layer spacing, as depicted in Fig. 1e. The specific surface area (SSA) of the prepared r-GO was 170.98 m^2^ g^−1^, while the SSAs were 209 and 89 m^2^ g^−1^ for CNTs and g-C_3_N_4_ nanosheets, respectively. Spectroscopic measurements were conducted using four filters over a 15-day period. The techniques employed were EDX, ICP, and LIBS.

### EDX technique results

**Figure 2:**
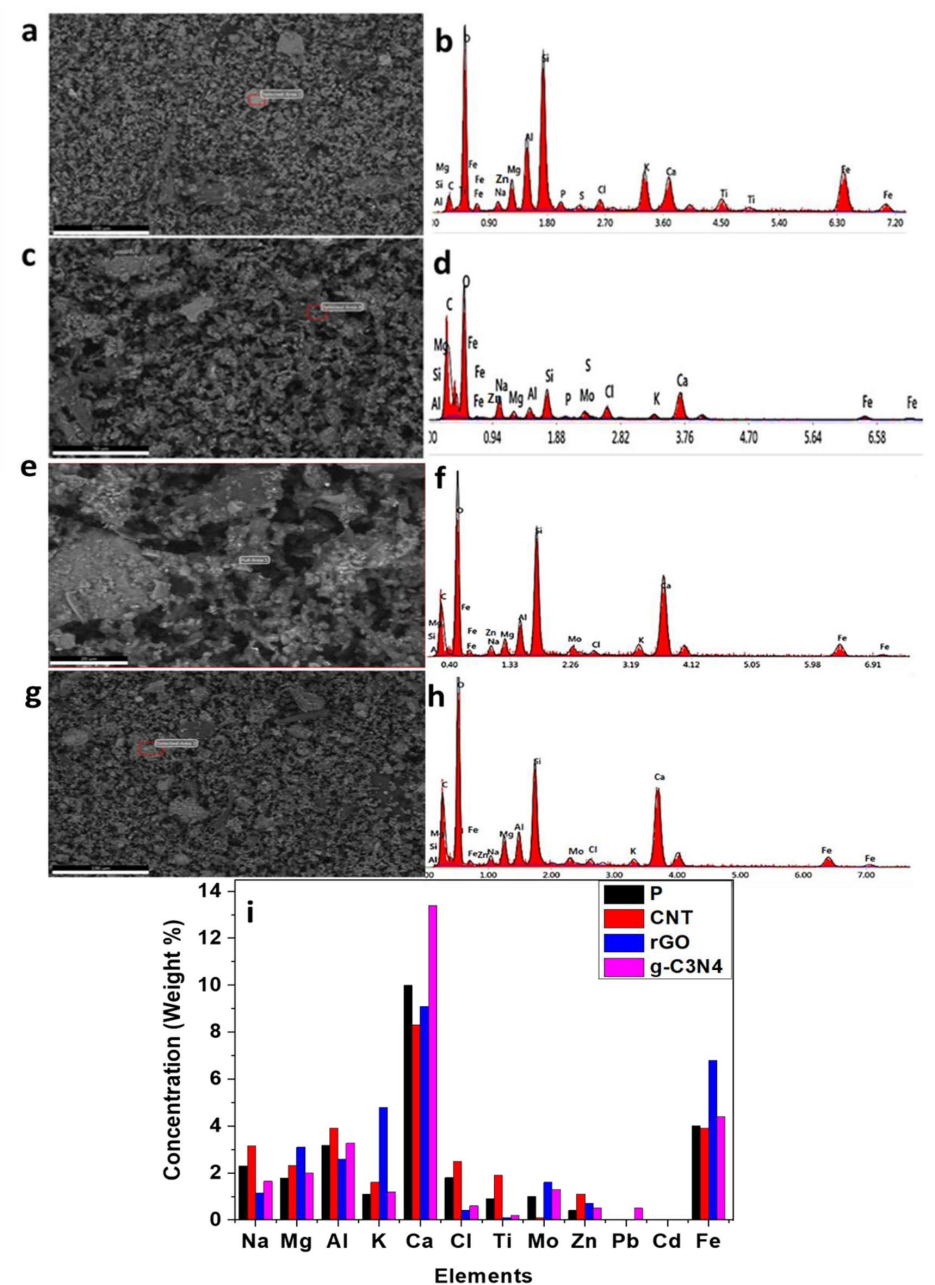
Overall (wt%) of the PMs for blank filter in comparison to the nano-modified filters with (CNTs, r-GO, and g-C_3_N_4_).

The morphology of the filter (P) was examined through SEM images and EDX analysis, as presented in Fig. 2. Various elements, including sodium, magnesium, aluminium, silicon, potassium, calcium, and iron, were found in the filter, with silicon being the most abundant element and titanium the least (Fig. 2a,b). These findings prompted further investigation into the presence of these elements in the other filters, aiming to monitor the impact of injected nanomaterials on the filters’ adsorption capacity for heavy elements and air pollutants. In Fig. 2c,d, an improve in the concentrations of potassium, molybdenum, iron, and magnesium elements was observed in this filter compared to blank filter. The percentage of magnesium, potassium, iron, Zinc, and molybdenum in the blank filter (1.78, 1.24, 3.76, 0.4, and 0.95 wt%, respectively) increased to (3.1, 4.77, 6.8, 1.7, and 1.6 wt%) in the filter injected with CNTs material, respectively. This indicates that the efficacy of the filter improves when injected with r-GO than the blank filter, leading to enhanced adsorption of suspended pollutants in the air sample. Figure 2e,f presents the SEM image and EDX spectrum of the filter blank with CNTs It is observed that the concentrations of aluminium, sodium, and titanium elements are higher in this filter compared to the blank filter. This indicates that the efficacy of the filter improves when injected with CNTs, leading to increased adsorption efficiency for suspended pollutants in the air sample. The percentage of titanium, sodium, and aluminium in the blank filter (0.9, 2.3, and 3.18 wt%, respectively) increased to (1.9, 3.15, and 3.91 wt%) in the filter injected with CNTs material, respectively. Lead was also not detected. Figure 2g,h presents the SEM image and EDX spectrum of the filter blank with g-C_3_N_4_. It is observed that the concentrations of calcium and iron elements are higher in this filter compared to filter P. This indicates that the efficacy of the filter improves when injected with g-C_3_N_4_, leading to increased adsorption efficiency for suspended pollutants in the air sample. The percentage of molybdenum, calcium, magnesium, and iron in the blank filter (0.95, 10.03, 1.78, and 3.76 wt%, respectively) increased to (1.34, 13.36, 2.17, and 4.36 wt%) in the filter injected with g-C_3_N_4_ material, respectively. Lead was not detected in the analysis of the first filter, but it appeared at a percentage of 0.54 wt% in the filter injected with g-C_3_N_4_ nanoparticles. They also exhibited improved efficiency in detecting and adsorbing PMs like lead. In general, the filters modified with carbon nanomaterials demonstrated superior performance compared to the blank filters in terms of their ability to adsorb particulate matters (Fig. 2i).

### ICP results

**Figure 3:**
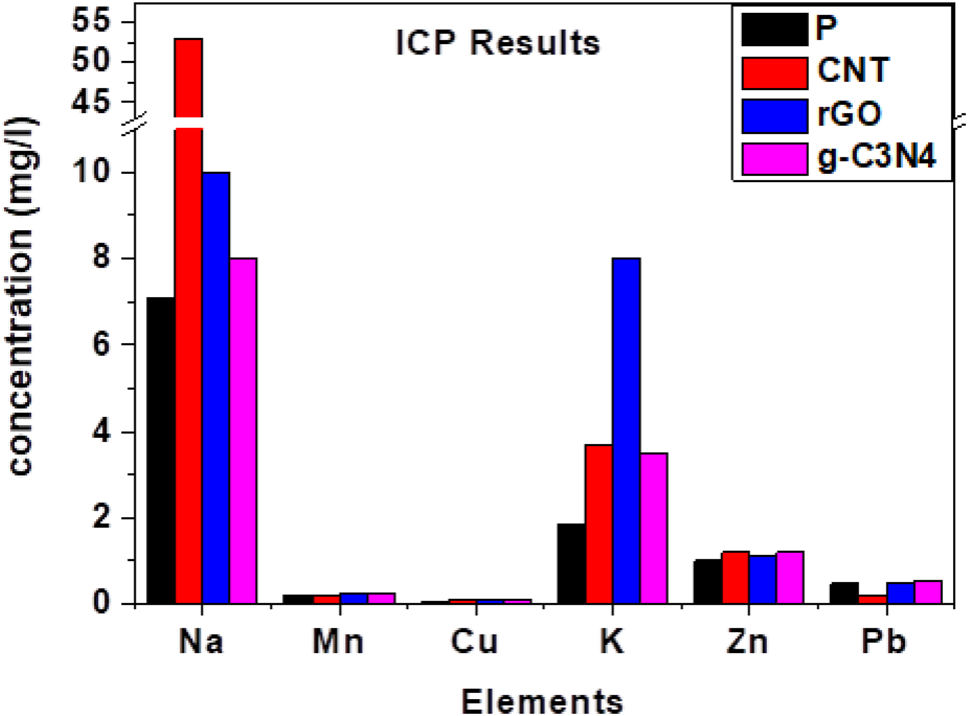
ICP results for the blank filter and the nano-modified filters.

During a 15-day period, four filters were operated at the same location as the initial three filters to observe any variations. Notable differences emerged when the filters underwent ICP analysis, which is detailed in Fig. 3. The Table 1 illustrates that the concentrations of lead, sodium, potassium, and manganese increased when different nanomaterials were used compared to the blank filter. It is worth mentioning that zinc was not detected during the EDX analysis but was detected when the ICP technique was employed.

**Table 1:** ICP Results for 15 operating days for the blank and carbon nano-modified filters.

Filter	Injected material	Injected material concentration	Cu (mg/l)	Na (mg/l)	Mn (mg/l)	Zn (mg/l)	Pb (mg/l)	K (mg/l)
Filter1	Nil	Nil	0.07	7.1	0.2	1	0.47	1.87
Filter2	CNTs	150 mg/l	0.08	53	0.19	1.2	0.2	3.7
Filter3	r-GO	150 mg/l	0.09	10	0.25	1.1	0.47	8
Filter4	g-C_3_N_4_	150 mg/l	0.09	8	0.25	1.2	0.55	3.5

### LIBS results

LIBS is a recently developed analytical technique that involves exciting the atoms in a sample without selectivity. This is achieved by directing a pulsed laser beam onto the sample’s surface, causing a small quantity of material (a few nanograms) to be ablated, atomized, and ionized, ultimately producing a plasma plume. The formation of plasma is a complex process influenced by the physical properties of the sample and experimental conditions, impacting factors such as photon absorption, stress wave formation, shock wave generation, melting, vaporization, laser shielding, and more. As the plasma forms, it begins to cool within microseconds, emitting both continuum and discrete radiation. The discrete transitions of excited atoms or ions in the plasma generate a characteristic spectral pattern, which allows for the identification of elements present in the sample. The emitted light spectrum is collected, fed into a spectrometer, and analysed to obtain information about the composition of the material. LIBS offers several advantages as an analytical technique. It can be used with solids, gases, and liquids, regardless of their conductivity. The sample preparation required is minimal or nonexistent, reducing the time consumed in the experiment. Since only a small quantity of material is ablated, LIBS can be considered partially non-destructive. Furthermore, LIBS has demonstrated the ability to analyse extremely hard materials such as ceramics and superconductors, and it enables simultaneous multi-elemental analysis. But there are also some disadvantages associated with LIBS. It can be relatively expensive, and obtaining suitable standards for quantitative analysis can be challenging. The multielement nature of LIBS introduces a large interference effect known as the matrix effect. Precision can be poor, typically ranging from 5 to 10%, depending on factors such as sample homogeneity, sample matrix, and laser excitation properties. To analyse the concentration of different elements using LIBS, the spectra of various positions within each filter sample are averaged to obtain a single spectrum for each filter. To minimize experimental fluctuations, the spectra are normalized using the Carbon line at 247 nm. A spectral line is selected for each element of interest, and the intensities of the elemental lines at their respective spectral lines (e.g., Na I at 589.5 nm, Mn I at 403.3 nm, Mg I at 280.2 nm, Fe I at 358 nm, Cu I at 327.3 nm, Cd I at 326.1 nm, Ti I at 506.4 nm, Al I at 309.2 nm, and Zn I at 330.2 nm) are tracked as indicators of elemental concentration in each filter.

**Figure 4:**
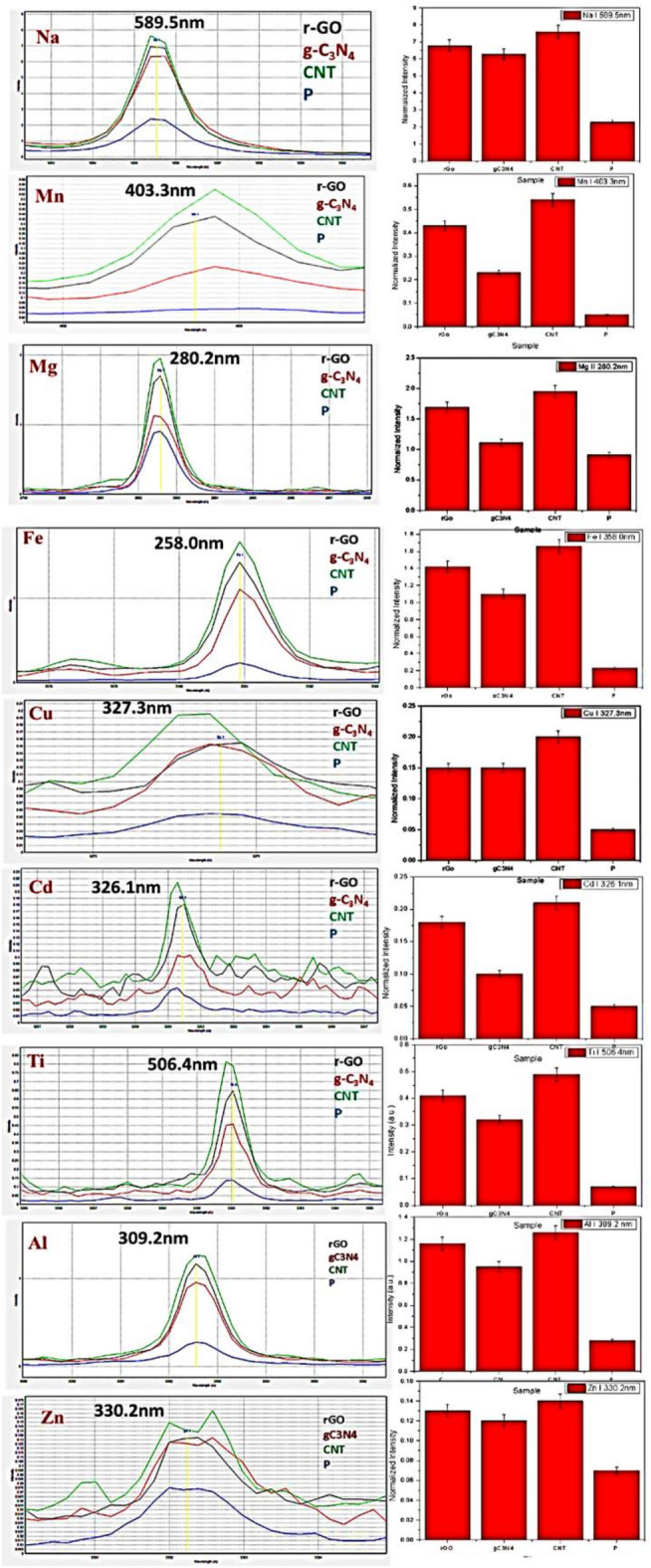
PMs precipitated at the filters with different treatments and their histogram representing the intensities of the PMs I spectral line.

**Figure 5:**
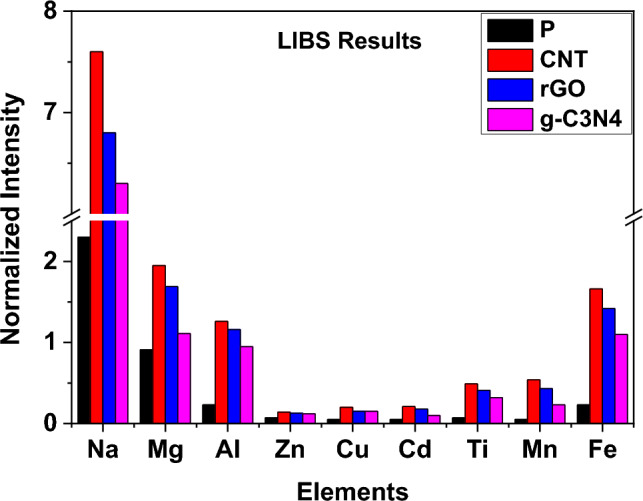
Overall LIBS Technique results for the blank and carbon nano-modified filters.

The histograms of all PMs reveal that the filter treated with CNTs exhibited the highest intensity, followed by the filter treated with r-GO, and then the filter treated with g-C_3_N_4_. The untreated filter (filter sample P) displayed the lowest intensity. It is evident that CNTs was the most effective carbon nanomaterial in improving the filter’s performance. The concentrations of the elements under study (sodium, manganese, magnesium, iron, copper, cadmium, titanium, aluminium, and zinc) were highest in the filter injected with CNTs, followed by the filter injected with r-GO. The filter injected with g-C_3_N_4_ showed lower concentrations of these elements. Conversely, the untreated filter without any nanomaterials exhibited the lowest concentrations (refer to Table 2 and Figs. 4, 5).

**Table 2:** Overall LIBS Technique results.

Element	Normalized intensity			
	P	CNT	r-GO	g-C_3_N_4_
Na	2.3	7.6	6.8	6.3
Mg	0.91	1.95	1.69	1.11
Al	0.23	1.26	1.16	0.95
Zn	0.07	0.14	0.13	0.12
Cu	0.05	0.2	0.15	0.15
Cd	0.05	0.21	0.18	0.1
Ti	0.07	0.49	0.41	0.32
Mn	0.05	0.54	0.43	0.23
Fe	0.23	1.66	1.42	1.1

There is a big difference between the LIBS spectral lines intensities in case of the treated and untreated samples. This may be attributed to, beside the low adsorption of elements in the untreated filters; the LIBS signal is enhanced when used with nanoparticles^24–26^.

### Comparison LIBS results to the ICP results

**Figure 6:**
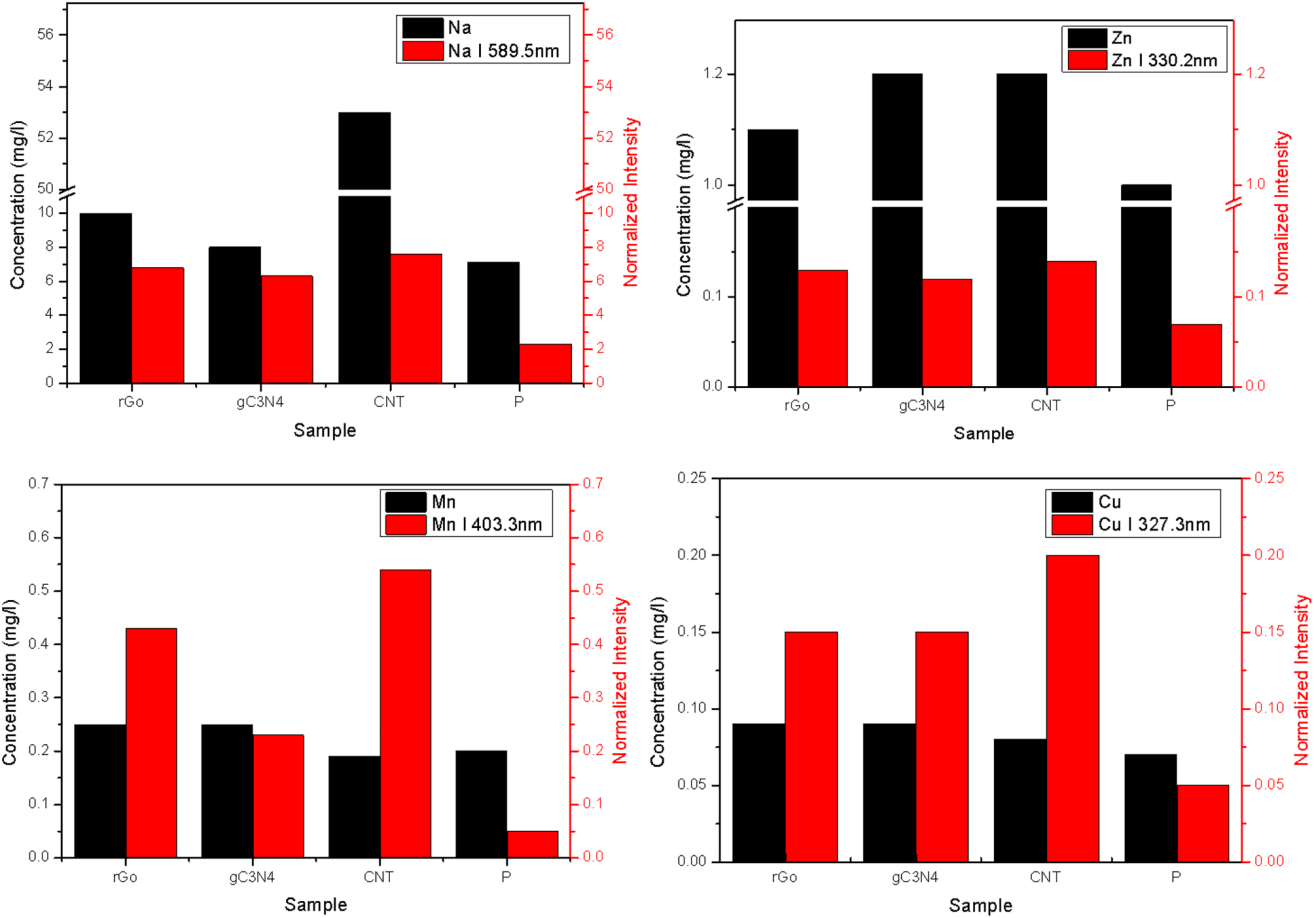
Comparison between LIBS results and ICP results for Na, Mn, Zn, and Cu PMs.

To justify the results obtained from LIBS, a comparison was conducted between the LIBS results and the results obtained from ICP analysis for the elements Mn, Na, Zn, and Cu. Figure 6 illustrates this comparison for the PMs Na, Mn, Zn, and Cu. The comparison reveals a strong agreement between the LIBS and ICP results for Na, Zn, and Cu. However, for Mn, there is some discrepancy between the two techniques. Both LIBS and ICP indicate a minimum value for the untreated filters. While ICP suggests that the filters treated with r-GO and CNT have the same concentration, LIBS indicates that the filters treated with r-GO exhibit a higher value than those treated with CNT for Mn. Additionally, LIBS shows that the highest intensity is observed in the filters treated with CNT (Fig. 6).

This comparison highlights that the filter injected with CNT material is the most effective in absorbing heavy elements from the withdrawn air sample. Although LIBS results align with ICP results, which indicate an increase in filter efficiency when nanomaterials are used compared to the filter without nanomaterials, LIBS alone cannot solely demonstrate this change in efficiency. This limitation arises because the LIBS signal is naturally enhanced by the presence of nanoparticles, as mentioned in previous studies^24–26^.

## Discussion

The comparison of results obtained from LIBS and ICP for the elements Mn, Na, Zn, and Cu yields valuable insights into the reliability and performance of the LIBS technique. In general, there is a substantial agreement between the LIBS and ICP outcomes for Na, Zn, and Cu, indicating that LIBS is a dependable method for analyzing the concentrations of these elements in the filter samples. However, some disparities are observed in the results for Mn. Both LIBS and ICP demonstrate a minimal value for the untreated filters, suggesting a lower Mn concentration in these samples. Interestingly, while ICP indicates that the filters modified with r-GO and CNT have the same Mn concentration, LIBS reveals a higher value for the filters modified with r-GO compared to those treated with CNT. Additionally, LIBS shows that the highest intensity is recorded in the filters modified with CNT. These inconsistencies raise questions about the accuracy and precision of the LIBS technique specifically for Mn analysis. The superior performance of the filter injected with CNT in absorbing heavy elements from the air sample is clearly demonstrated by both LIBS and ICP results. This finding highlights the potential of CNT as an effective nanomaterial for enhancing the efficiency of filters in capturing and retaining heavy elements. In conclusion, while LIBS shows good agreement with ICP results for Na, Zn, and Cu, further investigation is required to reconcile the discrepancies observed in the Mn analysis.

Carbon nanotubes, being the most common carbon allotrope, exhibit unique structural characteristics that make them effective for removing heavy metals from various environments. Their high active surface area to volume ratio and accurate aperture size measurements contribute to their exceptional adsorption capabilities, as demonstrated in the modified filters with CNTs. In comparison to conventional powdered and granular activated carbon, which have limitations such as smaller active surface areas and higher activation energy of adsorption, carbon nanotubes offer significant advantages in terms of adsorption potential and efficacy^27^.

Carbon-based particles are emerging as alternatives to CNTs for heavy metal removal, owing to their remarkable qualities such as excellent mechanical strength, electrical properties, and thermal conductivity. These materials, including reduced and oxide graphene, possess several advantages as adsorbents. They can serve as substrates for functionalization with groups that enhance metal ion sorption. During the adsorption process, the adsorbate accumulates on the surface of the adsorbent, primarily due to the partial absence of surrounding atoms. Unlike bulk materials, where other atoms within the substance fulfill the bonding requirements of the component atoms through ionic, covalent, or metallic bonds, the surface atoms of the adsorbent attract adsorbates. The bonding between adsorbates and the adsorbent surface involves weak van der Waals forces in the case of physisorption, or covalent bonds in the case of chemisorption. The specific nature of bonding depends on the species involved, and electrostatic attraction can also play a role^28,29^.

The mechanism of heavy metal removal by CBNMs involves various processes, including physical adsorption: Heavy metal ions are attracted to the surface of the CBNMs due to van der Waals forces. Chemical adsorption: Heavy metal ions form chemical bonds with the functional groups on the surface of the CBNMs. Ion exchange: Heavy metal ions exchange with other cations present on the surface of the CBNMs. Chelation: Heavy metal ions are complexed by organic molecules attached to the surface of the CBNMs. The findings of this study agree with the physical adsorption mechanism.

The effectiveness of CBNMs for heavy metal removal depends on various factors, including the type of CBNM, the surface area and functional groups, the type and concentration of heavy metal ions. CBNMs offer several advantages over traditional methods for heavy metal removal: Higher efficiency: CBNMs can remove heavy metals with higher efficiency than conventional methods. Selectivity: CBNMs can be tailored to selectively remove specific heavy metals. Reusability: CBNMs can be easily regenerated and reused multiple times, reducing the cost and environmental impact. Environmentally friendly: CBNMs are generally considered to be environmentally friendly and biodegradable.

Despite the promising potential of CBNMs, some challenges remain to be addressed, such as: Cost: The production of some CBNMs can be expensive. Scalability: The large-scale production and application of CBNMs for heavy metal removal need further development. Environmental impact: The potential environmental risks of CBNMs need to be further evaluated.

Overall, CBNMs offer a promising and sustainable solution for the removal of heavy metals from contaminated water and wastewater. As research and development continue, CBNMs are expected to play an increasingly important role in environmental remediation efforts.

## Conclusions

In conclusion, air pollution, particularly particulate matter, poses significant threats to human health and the environment. The incorporation of carbon nanostructured materials into air particle monitoring devices holds great potential for enhancing the efficiency of particulate matter adsorption. The utilization of g-C_3_N_4_ NSs, r-GO, and CNTs as nanostructures for modifying filters shows promise for improving air quality and reducing the harmful effects of air pollution. This study aims to investigate the impacts of these nanomaterials on filter performance and explore the potential of LIBS for sensitive heavy metal monitoring. The outcomes of this research will contribute to the development of innovative strategies for air pollution remediation and the protection of human health and the environment.

## Materials and methods

### Synthesis of carbon nanomaterials

#### Synthesis of r-GO

The synthesis of GO was conducted using a modified version of Hummer’s method^30^. To obtain r-GO from GO, 100 mg of the dry GO powder was placed in an empty beaker. The beaker was then positioned on a hot plate set at 350 °C for 10 min, with an aluminium foil cover that had several perforated pores, placed over it. The resultant was a black powder of r-GO, which was carefully removed from the beaker^31,32^.

#### Synthesis of CNTs

The fabrication of CNTs was achieved through the utilization of nonporous anodic alumina membranes (NAAMs) in a template-free and catalyst-free chemical vapor deposition (CVD) synthesis method. The two-stage furnace used for this procedure included a cylindrical quartz tube within that was 4.3 cm long and 100 cm in diameter. The furnace was designed to provide precise control over gas flow rates during the synthesis of CNTs^23^.

#### Synthesis of g-C_3_N_4_

The synthesis of g-C_3_N_4_ involved heating 50 g of urea in air at a temperature of 550 °C for a duration of 4 h^15^.

### Preparation of nano-modified filters

Microfiber filters made of extremely pure quartz (SiO_2_) are suitable for PM10 testing and air sampling in environments with acidic gases, stacks, flues, and aerosols, especially when exposed to temperatures up to 500 °C. These filters exhibit minimal presence of “artefact” products of sulphates and nitrates (formed from SO_2_ and NO_2_) due to the low concentration of alkaline earth metals (Table 3). The filters, labelled as QM-A and numbered according to the standards set by the United States Environmental Protection Agency (EPA), are widely applicable and suitable for various purposes^30^.

**Table 3:** General description of filter paper.

Grade	QM-A
Description	Quartz
Particle Retention in Liquid (μm)	2.2
Air Flow (s/100 mL/in^2^)	6.4
Typical Thickness (μm)	450
Basis Weight (g/m^2^)	85

**Figure 7:**
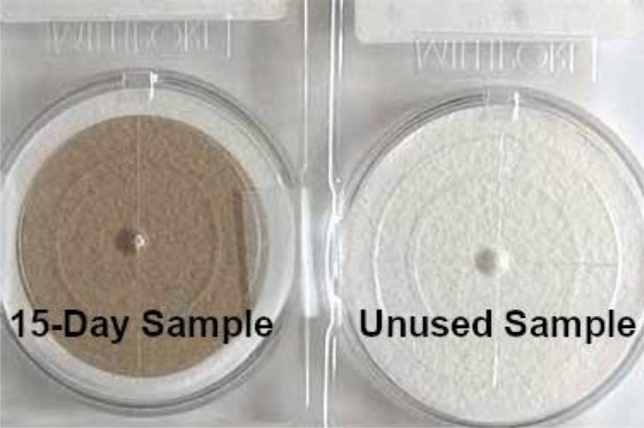
Images for the nano-modified filters before and after air sampling.

Take 20 mg of the nanomaterial and place it in a 100 mL measuring flask. Fill the flask to the mark with ethanol and dissolve the contents using an oscillator for 15 min to obtain a stock solution with a concentration of 200 ppm. From this stock solution, you can prepare any desired concentration by dilution. Before use, weigh the filter and then inject it with 1 mL of the required concentration of the nanomaterial solution using a micro-pipette. Place the filter in a hood with suction for half hour to allow it to dry, and then weigh it again. Once weighed, the filter is ready for use with the air sampler instrument (Fig. 7). Ensure optimum protection against hazardous substances by utilizing a fume hood. Prior to impregnation and after impregnation and sampling, use a balance to weigh the filters. Utilize a cabinet to ensure complete drying of the filters before operation. After digestion, filter the operated filters using double ring filter paper. Finally, save the solution after filtration and even for analysis in an ICP using a Polypropylene Conical tube.

### Operation of air sampler’s instrument: general air-sampling methodology

Place the filter in the holder and securely install the holder into the air sampling instrument. Start the instrument and allow it to run for the specified time. Once the sampling is complete, remove the holder from the instrument and carefully take out the filter. Proceed with the chemical or spectroscopic analysis of the filter.

This study was conducted with parallel samples and the MiniVol™ Tactical Air Sampler (TAS) was used which is a portable ambient air sampler for particulate matter that can also be configured for sampling various air toxics. The patented low flow technology used in the MiniVol™ TAS was developed jointly with the U. S. Environmental Protection Agency (EPA) to address the need for portable air pollution sampling technology. While not a reference method sampler, the mass concentrations of the MiniVol™ TAS gives results that closely agree with reference method concentrations. Both accurate and precise, the battery operated, lightweight MiniVol™ TAS is ideal for sampling at remote sites or areas without power. In the particulate matter (PM) sampling mode, air is drawn through a particle size separator and then through a filter medium. Particle size separation is achieved by impaction. Critical to the collection of the correct particle size is the correct flow rate through the impactor. For the MiniVol™ TAS, the actual volumetric flow rate must be 5 litters per minute (5 lpm) at ambient conditions.

### Chemical analysis of filters

The four utilized filters underwent analysis using ICP to determine the concentration of elements present on each filter. This analysis was conducted to conduct a comprehensive study of each filter and perform a qualitative analysis of the dust accumulated on them. These results aimed to provide insights into the impact of nanomaterials on the filters and evaluate their efficiency in capturing particulate matter.

### Air matrix sampler

Sampling devices based on pumps inherently cannot achieve 100% efficiency in collecting particles within a specific size range. For instance, particle size selection devices like the Well Impactor Ninety-Six (WINS) or Very Sharp Cut Cyclone (VSCC), commonly used in PM2.5 sampling, are designed to collect approximately 50% of particles with a 2.5 µm aerodynamic diameter while allowing the remaining 50% to pass through the device. The collection efficiency of these devices improves for particles larger than 2.5 µm, while particles smaller than 2.5 µm pass through the device with higher penetration efficiency until they are finally collected on a collection plate or filter.

### Characterization of nanomaterials

Characterization of the nanomaterials involved various techniques. Scanning electron microscopy (SEM) images were obtained using a ZEISS FE-SEM ULTRA Plus microscope equipped with an EDX analyzer and a Philips CM20 microscope, operating at an accelerating voltage of 200 kV. Sample dispersion was deposited onto an aluminium pin stub and left to evaporate at room temperature^33^. X-ray diffraction (XRD) measurements were conducted using a Philips PW1710 X-ray diffractometer with Cu Ka radiation (k = 1.54186 A°). The XRD patterns were recorded within the 20° to 70°2Ɵ range with a step size of 0.020°2Ɵ and a collection time of 10 s per step. FT-IR spectra were recorded using a Nicolet 6700 infrared spectrophotometer to identify specific functional groups present on the surface. Based on the Brunauer–Emmett–Teller (BET) model, the precise surface area and pore volume were calculated using Micromeritics ASAP 2010 to collect N_2_ sorption isotherms. The samples were first outgassed under vacuum at 60 °C overnight, before being examined at 77 K.

### LIBS experimental procedures

For the experimental part of the work, plasma was generated by focusing a 50 mJ Nd: YAG laser with a pulse duration of 5 ns (Brio, Quantel, France) onto the surface of the filter under study. The laser operated at a fundamental wavelength of 1064 nm in air at atmospheric pressure, and the plasma emission was collected by a quartz optical fiber with a 600 µm diameter aperture. The optical fiber’s aperture was aligned, and the distance between the fiber and plasma plume was adjusted to ensure that the fiber covered most of the plume emission, minimizing inhomogeneity in the laser-induced plasma. The output of the optical fiber was connected to an echelle spectrometer coupled to an intensified charge-coupled device (ICCD) (Mechelle 7500, DiCAM–Pro system), enabling simultaneous spectral analysis in the range of 200–700 nm with a constant spectral resolution (λ/Δλ = 7500). A delay of 1500 ns and a gate width of 2000 ns were chosen to maximize spectral line intensity. The spectrometer’s gate width and delay time were controlled by a computer. To optimize signal-to-noise ratio and spectral reproducibility, 10 single spectra were accumulated from different positions on the sample surface. Additionally, to mitigate sample inhomogeneity, the average spectra of five different accumulated spectra were taken for each measurement. To minimize angular dispersion of the plasma emission, measurements were performed at distances from the target surface that were shorter or comparable to the spot dimension. The emission spectra were analysed using the LIBS++ software^34^. The measurements were repeated under the same conditions for each filter.

## Data Availability

All data generated or analyzed during this study are included in this published article.

## References

[1] Ghosh N, Goyal S, Howard A, Banerjee P, Vitale J (2023). Application of nanotechnology in a novel air purifier for remediation of airborne pathogen and to prevent the spread of COVID-19. Eur. Sci. J..

[2] Mahmoudi A, Tavakoly Sany SB, Ahari Salmasi M, Bakhshi A, Bustan A, Heydari S, Rezayi M, Gheybi F (2023). Application of nanotechnology in air purifiers as a viable approach to protect against Corona virus. IET Nanobiotechnology.

[3] Kumar, R., Gupta, K. & Bordoloi, N. Nanotechnology: An emerging strategy for combating air pollution. In *Environmental Sustainability and Industries* (eds Singh, P., Bassin, J. P., Rajkhowa, S., Hussain, C.M. & Oraon, R.) 117–128. 10.1016/B978-0-323-90034-8.00017-8 (Elsevier, 2022).

[4] Mukherjee A, Agrawal M (2017). World air particulate matter: Sources, distribution and health effects. Environ. Chem. Lett..

[5] MacMurdo MG, Mulloy KB, Felix CW, Curtis AJ, Ajayakumar J, Curtis J (2022). Ambient air pollution exposure among individuals experiencing unsheltered homelessness. Environ. Health Perspect..

[6] Argyropoulos CD, Hassan H, Kumar P, Kakosimos KE (2020). Measurements and modelling of particulate matter building ingress during a severe dust storm event. Build Environ..

[7] Badaloni C, Cesaroni G, Cerza F, Davoli M, Brunekreef B, Forastiere F (2017). Effects of long-term exposure to particulate matter and metal components on mortality in the Rome longitudinal study. Environ. Int..

[8] Brauer M, Brook JR, Christidis T, Chu Y, Crouse DL, Erickson A, Hystad P, Li C, Martin RV, Meng J, Pappin AJ, Pinault LL, Tjepkema M, van Donkelaar A, Weagle C, Weichenthal S, Burnett RT (2022). Mortality-air pollution associations in low exposure environments (MAPLE): Phase 2. Res. Rep. Health Eff. Inst..

[9] Espinosa R, Palma J, Jiménez F, Kamińska J, Sciavicco G, Lucena-Sánchez E (2021). A time series forecasting based multi-criteria methodology for air quality prediction. Appl. Soft Comput..

[10] Saleem H, Zaidi SJ, Ismail AF, Goh PS (2022). Advances of nanomaterials for air pollution remediation and their impacts on the environment. Chemosphere.

[11] Lyu C, Zhao P, Xie J, Dong S, Liu J, Rao C, Fu J (2021). Electrospinning of nanofibrous membrane and its applications in air filtration: A review. Nanomaterials.

[12] Keshta AT, Fathallah AM, Attia YA, Salem EA, Watad SH (2023). Ameliorative effect of selenium nanoparticles on testicular toxicity induced by cisplatin in adult male rats. Food Chem. Toxicol..

[13] Kolosnjaj-Tabi J, Szwarc H, Moussa F (2017). Carbon nanotubes: Culprit or witness of air pollution?. Nano Today.

[14] Khan, A., Malik, S., Ali, N., Nguyen, T.A. & Bilal, M. Nanoadsorbents as a green approach for removal of environmental pollutants. In *Micro and Nano Technologies, Nano-Bioremediation: Fundamentals and Applications* (eds Iqbal, H. M. N., Bilal, M. & Nguyen, T. A.) 435–454 (Elsevier, 2022).

[15] Abdelsalam EM, Samer M, Seifelnasr A, Moselhy MA, Ibrahim HHA, Faried M, Attia YA (2023). Effects of Al_2_O_3_, SiO_2_ nanoparticles and g-C_3_N_4_ nanosheets on bio cement production from agricultural wastes. Sci. Rep..

[16] Janani R, Gurunathan B, Sivakumar K, Varjani S, Ngo HH, Gnansounou E (2022). Advancements in heavy metals removal from effluents employing nano-adsorbents: Way towards cleaner production. Environ. Res..

[17] Mohamed YMA, Attia YA (2023). Nano Pt/TiO_2_ photocatalyst for ultrafast production of sulfamic acid derivatives using 4-nitroacetanilides as nitrogen precursor in continuous flow reactors. Environ. Sci. Pollut. Res..

[18] Abdelsalam EM, Mohamed YMA, Abdelkhalik S, El Nazer H, Attia YA (2020). Photocatalytic oxidation of nitrogen oxides (NO_x_) using Ag- and Pt-doped TiO_2_ nanoparticles under visible light irradiation. Environ. Sci. Pollut. Res..

[19] Mammadova S, Nasibova A, Khalilov R, Mehralıyeva S, Valiyeva M, Gojayev AS, Zhdanov RI, Eftekhari A (2022). Nanomaterials application in air pollution remediation. Eurasian Chem. Commun..

[20] Kumar S (2023). Carbon based nanomaterial for removal of heavy metals from wastewater: A review. Int. J. Environ. Anal. Chem..

[21] Baby R, Saifullah B, Hussein MZ (2019). Carbon nanomaterials for the treatment of heavy metal-contaminated water and environmental remediation. Nanoscale Res. Lett..

[22] Mallakpour S, Khadem E, Kyzas GZ, Mitropoulos AC (2019). Carbon nanotubes for heavy metals removal. Micro and Nano Technologies, Composite Nanoadsorbents.

[23] Attia YA, Al Nazawi AM, Elsayed H, Sadik M (2021). Carbon nanotubes catalysed UV-trigger production of hyaluronic acid from *Streptococcus equi*. Saudi J. Biol. Sci..

[24] De Giacomo A, Dell’Aglio M, Gaudiuso R, Koral C, Valenza G (2016). Perspective on the use of nanoparticles to improve LIBS analytical performance: Nanoparticle enhanced laser induced breakdown spectroscopy (NELIBS). J Anal. At. Spectrom..

[25] Dell’Aglio M, Alrifai R, De Giacomo A (2018). Nanoparticle enhanced laser induced breakdown spectroscopy (NELIBS) a first review. Spectrochim. Acta B.

[26] Abdelhamid M, Attia YA, Abdel Harith M (2020). Significance of the nano-shapes in nanoparticle-enhanced laser-induced breakdown spectroscopy. J. Anal. At. Spectrom..

[27] Chandran G, Muruganandam L, Biswas R (2023). A review on adsorption of heavy metals from wastewater using carbon nanotube and graphene-based nanomaterials. Environ. Sci. Pollut. Res..

[28] Ahmad SZN, Wan Salleh WN, Ismail AF, Yusof N, Mohd Yusop MZ, Aziz F (2020). Adsorptive removal of heavy metal ions using graphene-based nanomaterials: Toxicity, roles of functional groups and mechanisms. Chemosphere.

[29] Xu X, Zeng J, Wu Y, Wang Q, Wu S, Gu H (2022). Preparation and application of graphene-based materials for heavy metal removal in tobacco industry: A review. Separations.

[30] Attia YA (2016). Ag/ZnO/graphene-TBSCl hybrid nanocomposite as highly efficient catalyst for hydrogen production. Mater. Express.

[31] Wang B, Zhang F, He S, Huang F, Peng Z (2014). Adsorption behaviour of reduced graphene oxide for removal of heavy metal ions. Asian J. Chem..

[32] Alam SN, Sharma N, Kumar L (2017). Synthesis of graphene oxide (GO) by modified hummers method and its thermal reduction to obtain reduced graphene oxide (rGO). Graphene.

[33] Stokes DJ (2008). Principles and Practice of Variable Pressure Environmental Scanning Electron Microscopy.

[34] Cremers DA, Radziemski LJ (2013). Handbook of Laser Induced Breakdown Spectrometry.

